# Effective infection prevention and control strategies in a large, accredited, psychiatric facility in Singapore

**DOI:** 10.1017/ice.2020.163

**Published:** 2020-04-23

**Authors:** Daniel Poremski, Sandra H. Subner, Grace F.K. Lam, Raveen Dev, Yee Ming Mok, Hong Choon Chua, Daniel SS Fung

**Affiliations:** 1 Institute of Mental Health, Singapore, Singapore; 2 National Healthcare Group, Singapore, Singapore

*To the Editor—* On January 14, 2020, Singapore, a population-dense equatorial island nation in Asia, experienced its first COVID-19 case. The spread of SARS-CoV-2 reached 100 people over the first 6 weeks of the infection.^1^ The country took measures to reduce the porosity of its borders and implemented special measures to limit community transmission without immediately closing schools and businesses.^1,2^ Because infections may spread quickly in psychiatric facilities,^3,4^ special measures were introduced in such settings. Although the cultural setting and geographical location might be unique, Singapore’s Institute of Mental Health (IMH) operations are similar to those of international medical facilities accredited by The Joint Commission. This structure lends to the generalizability of several of its operational strategies. Here we have summarized the steps taken at the IMH, Singapore’s largest provider of tertiary mental health care, which has, as of April 28, 2020, prevented the spread of SARS-CoV-2 despite local community transmission.^1^

The IMH serves this nation’s population of ~6 million; it employs ~2,500 staff and has a capacity of 2,000 inpatient beds. Occupancy reaches 51,000 patient bed days per month. The IMH receives ~16,000 emergency service visits^5^ and 36,000 outpatient visits, and it hosts 20,000 family visitors to inpatient wards each year. It is also a primary location for teaching. In 2005, the IMH was the first mental health institute to obtain Joint Commission International (JCI) accreditation in Asia.

The architecture of the 22-hectare campus emphasizes natural light and air circulation through every general ward year-round, made possible by the equatorial climate. The facility has 28 JCI-compliant high-efficiency particulate air (HEPA)–filtered, negative-pressure, medical isolation units, and 14 of these have additional anterooms. Old and new administration buildings allow administrative departments to be physically split between buildings. Because standard infection prevention practices may not invariably prevent the spread of infections,^4,6-8^ additional measures were implemented to respond to the pandemic.

## Infection prevention and control strategies

### Providing essential mental health services

Providing mental health services is the mission of the IMH. However, certain aspects may need to be balanced to safeguard the sustained provision of services. First, care should always be provided with the least restrictive means. Finding this balance requires careful and constant assessment of risks and the effectiveness of current strategies. For example, completely suspending family visits may help reduce the risk of SARS-Cov-2 transmission considerably, but such a policy would be detrimental to the recovery of patients, and under the current risk assessment, it is a step too far.

The decision to reduce access to or suspend individual services entirely depends on (1) the risk of infection posed by the service, (2) the needs of patients, and (3) service alternatives. For example, outpatient services may introduce infection because physicians have inpatient and outpatient duties. Reducing the volume of outpatient services could help decrease risks. Patients with low levels of need are served via telehealth consultations. Medications are delivered by courier to reduce the volume of patients entering the facility.

### Preventing the introduction of infectious contagions

A guiding principle of the infection prevention and control strategies is to recognize that patients and staff need to be equally considered to ensure that infections are avoided. Several general prevention strategies are used under normal risk of infection,^9^ and they are considered normal accreditation obligations. Special strategies introduced to keep infectious contagions out of the facilities are listed in Table 1.

**Table 1.** Pandemic-Specific Infection Prevention and Control Strategies, Stratified by Level

Hospital	Ward/Location	Individual
Restricted access into IMH, only 1 entrance 3 visitor screening centers have been set up Visitors must register and state the purpose of their visit and location of visit Temperature screening of all visitors plus travel history Restrictions on the number of visitors: only 1 visitor at a time per inpatient; only 1 accompanying person per outpatient Televisitation services for visitors Teleconsultation for medical consultations in nursing homes Home delivery of prescriptions	Isolation ward for potential infectious cases (eg, respiratory symptoms) Enhanced pneumonia surveillance Modular system in the blocks; no cross-block movement; split-mode operations No interward mixing Unidirectional flow in the outpatient clinic Reduction of outpatient appointments to lengthen the gap between follow-up appointments where possible Negative pressure rooms (n=28) **ECT service** Segregation of inpatients and outpatients receiving service; service provision is conducted by blocks; enhanced terminal cleaning after each use **X-ray services** Provision of services by block **Dental services** Suspended service	**Patients** Temperature surveillance of inpatients twice daily Patients hands are sanitized every 2 h in the wards Personal hygiene education for inpatients Patient vaccination program (flu and pneumococcal) Suspension of group activities **Staff** Staff wear surgical masks in the wards Personal portable hand sanitizer given to all staff, changed every 6 mo Staff temperature screening twice daily Travel and leave restrictions Staff vaccination program (flu, MMR, hepatitis) All nonessential training has been suspended Essential training, ie, maintenance of competency (eg, BCLS) continues Personal protective equipment training and audits Electronic tracking of staff movement to facilitate contact tracing Monitoring of staff temperature, travel, and medical certificates using a national-level staff surveillance system **Visitors** Suspension of all volunteer activities All visitors to wards must wear masks

Note. IMH, Singapore Institute for Mental Health; ECT, electroconvulsive therapy; MMR, measles, mumps, rubella vaccine; BCLS, basic cardiac life support.

### Resource management

The sustainability of strategies is vital, especially when expecting a long pandemic. As such, policy makers must be mindful of the supply of consumables and labor. Managing consumables may depend on local supply chains. In Singapore, a national-level strategy ensures that personal protective equipment is available. Allocation is monitored down to the individual mask. This measure ensures that the demand for consumables can be quantified precisely.

Business continuity planning is a priority. To avoid quarantine of entire departments, each department has been split into individual units that conduct all the essential functions of the larger department. This isolates the departments similar to the encapsulated operation of the wards.

To ensure that staff are available, nonessential vacation allowances have been suspended. Because suspended travel may incur financial costs, one of the first items communicated to staff included the Ministry’s intention to compensate staff for disruptions to personal travel. This action reduced anxiety and allowed personnel to focus on their duties.

Communication is vital. Regular updates are given to staff to inform them of essential developments, which are communicated via town halls, e-mails, and social media, depending on urgency. Senior management also increased the frequency of their presence in the wards to ensure staff engagement.

Recognition is important. Special compensation was announced for frontline staff. Although the actual reward for going above and beyond the call of duty may not come immediately, it is essential that staff be notified early that they will be recognized for their added effort.

In conclusion, infection prevention and control strategies come with varying degrees of immuration. Deploying a multipronged approach that addresses care and safety of staff and patients while underscoring the sustainability service provision is vital. It is imperative that healthcare organizations respond with overly cautious strategies and that they subsequently monitor the effect of these measures to balance the protection of patients with the tenet of providing the least restrictive service. In matters that are time sensitive, leaders should avoid fatiguing their staff with repeated policy changes because it will lead to complacency. The challenges of prolonged pandemics are felt by everyone, and ultimately, it is important to recognize and validate the contribution of individuals.
